# Study on the mechanism of action of *Scutellaria barbata* on hepatocellular carcinoma based on network pharmacology and bioinformatics

**DOI:** 10.3389/fphar.2022.1072547

**Published:** 2023-01-09

**Authors:** An-Yin Yang, Hong-Li Liu, Yong-Feng Yang

**Affiliations:** ^1^ Department of Liver Disease, Second Hospital of Nanjing, Nanjing University of Chinese Medicine, Nanjing, China; ^2^ Medical College of Southeast University, Nanjing, China

**Keywords:** hepatocellular carcinoma, network pharmacology, Scutellaria barbata, oral delivery, target validation, drug development

## Abstract

**Background:** Hepatocellular carcinoma is one of the most common cancers with the characteristics of invasion and high mortality. Current forms of prevention remain severe. *Scutellaria barbata* is widely used in traditional Chinese medicine treatment of various tumors. This study explored the mechanism of *Scutellaria barbata* in the treatment of hepatocellular carcinoma by network pharmacology and bioinformatics.

**Methods:** The active ingredients of *Scutellaria barbata* and potential targets for the treatment of hepatocellular carcinoma were collected by network pharmacology. The protein interaction network was constructed to screen the core targets, and the association between the core targets and diseases was further verified by bioinformatics methods. Finally, the active ingredients corresponding to the targets closely related to the disease were screened for AMDE characteristics analysis. Molecular docking of drug-like ingredients with corresponding targets was performed. We used CCK-8 kit to determine the effect of active ingredients on cell proliferation.

**Results:** 29 candidate active ingredients and 461 related targets of *Scutellaria barbata* were screened. A total of 8238 potential therapeutic targets for hepatocellular carcinoma were indentified. Finally, 373 potential targets for the treatment of HCC were obtained. The active ingredients: wogonin, Rhamnazin, eriodictyol, quercetin, baicalein, and luteolin, etc. The core targets were CDK1, CDK4, SRC, and E2F1. A total of 3056 GO enrichment entries were obtained, and 180 enrichment results were obtained by KEGG pathway analysis. Genes were mainly enriched in PI3K-Akt signaling pathway, IL-17 signaling pathway, TNF signaling pathway, apoptosis pathway, and hepatocellular carcinoma pathway. Molecular docking results showed that the screened compounds had strong binding ability with the corresponding target proteins. CCK8 assays showed that Rhamnazin and Luteolin suppressed the proliferation of HCC cells significantly compared with controls.

**Conclusion:** This study revealed that the mechanism of *Scutellaria barbata* in the treatment of hepatocellular carcinoma may be that the active ingredients inhibit the expression of core genes and block the PI3K-AKT signaling pathway to inhibit the proliferation, and migration and induce apoptosis of cancer cells.

## Introduction

Hepatocellular carcinoma (HCC) is a leading cause of cancer-related death in many parts of the world (Yang et al., 2019). Hepatectomy is a treatment option for patients with non-cirrhotic HCC who can undergo wide resection without life-threatening complications. Surgical resection is a potentially curative treatment, but almost 70% of patients develop recurrent HCC after resection (Tabrizian et al., 2015). In theory, liver transplantation is the best treatment option because it treats both the tumor and the underlying cirrhosis. Tumor ablation is a widely accepted treatment option for patients with early-stage HCC (Forner et al., 2018). Transarterial chemoembolization (TACE) is an effective treatment option in patients with intermediate stage HCC (Lencioni et al., 2016). Sorafenib was the first drug approved for first line systemic treatment of patients with advanced-stage HCC. Despite the recent progress in prevention and treatment technology, the long-term survival of liver cancer is still poor due to late diagnosis and tumor recurrence/metastasis (Yang et al., 2019). In general, the treatment of liver cancer is difficult, and the form of prevention and treatment of liver cancer is severe. It is important to developing new drugs for HCC treatment.

Chinese medicine is an important component of comprehensive treatment measures for malignant tumors. Its advantages include enhancing the immunity of the body, improving the quality of life of patients, prolonging the survival time of patients, and playing a dynamic role in the clinical treatment of HCC(Liu et al., 2017; Yan et al., 2017; Zhai et al., 2019). Traditional Chinese medicine (TCM) has the characteristics of “multi-ingredient, multi-target and multi-pathway”. Network pharmacology developed on the basis of the rapid development of system biology and computer technology is an important bridge to combine modern TCM with modern medicine. It provides a new tool and concept for evaluating the mechanism of drug action (Fang et al., 2017; Zhang et al., 2017; Liu et al., 2018). It breaks the traditional thinking mode and framework of “one drug, one target and one disease”, which is consistent with the overall concept of TCM treatment and the principle of dialectical treatment. It is a new model of TCM development. Drug discovery has important implications for cancer treatment and precision medicine. Traditional drug discovery methods are mainly based on *in vivo* animal experiments and *in vitro* drug screening, but these methods are usually costly and labor-intensive. Over the past decade, the explosion of omics data has created opportunities for computational anticancer drug prediction and improved the efficiency of drug discovery (Li et al., 2020). More over, Bioinformatics is playing an increasingly important role in nearly all aspects of drug discovery, evaluation, and development. This growing importance stems not only from the role of bioinformatics in processing large amounts of data, but also from the utility of bioinformatics tools in predicting, analyzing, or interpreting clinical and preclinical outcomes. Molecular docking can reveal the molecular mechanism of drug treatment of diseases, elevate pharmacological effects to molecular level, and lay the material foundation for drug mechanism research.

*Scutellaria barbata* has a long history of application. Pharmacological studies provide evidence for its anti-tumor, antioxidant, antibacterial, anti-inflammatory, antiviral and immune regulation activities. The main clinical indications are the treatment of sore throat, jaundice, hepatitis and adjuvant treatment of cancer (Chen et al., 2020). For example, TF-SB can inhibit the growth of a variety of tumor cells through several specific signaling pathways (Zheng et al., 2018). In this study, the active ingredients, targets and pathways of *Scutellaria barbata* on hepatocellular carcinoma were analyzed by network pharmacology and molecular docking technology.

## Methods

### Collection of active ingredients and potential targets of traditional Chinese medicine

All chemical constituents of *Scutellaria barbata* were searched in the TCMSP database (https://old.tcmsp-e.com/molecule.php?qn=1925). According to the pharmacokinetic parameters: oral bioavailability (OB) ≥ 30%, and drug-like index (DL) ≥ 0.18, the eligible candidate active ingredients were screened, and the related targets of the candidate active ingredients screened in the TCMSP database were collected. The selected candidate active ingredients were retrieved in Pubchem database (https://pubchem. ncbi. nlm. nih. gov/), and the SDF file with 2D structure was downloaded. Then, the Swiss Target Prediction database (http://www.swisstargetprediction.ch/) was imported to predict the target of active ingredients. Finally, the targets collected from the Swiss Target Prediction and TCMSP databases were merged, and the repetitive values were removed as the targets of candidate active ingredients of *Scutellaria barbata*.

## Collection of disease targets of hepatocellular carcinoma

The keywords “Hepatocellular carcinoma” and “HCC” were searched in the TTD database (https://db.idrblab.org/ttd), GeneCards database (https://www.genecards.org/), Disgenet database (http://www.disgenet.org/web/DisGeNET/menu/home), and OMIM database (https://www.omim.org/), and all selected potential therapeutic targets for hepatocellular carcinoma were combined to remove duplicate values as targets for hepatocellular carcinoma.

### Targets for drug treatment of diseases

The collected candidate active ingredient targets and disease targets were crossed as potential targets for the treatment of diseases.

## Construction of the PPI network and screening and validation of core genes

To clarify the interaction between active ingredients and targets. The target of *Scutellaria barbata* for disease treatment was imported into the STRING online platform (https://cn.string-db.org/), the protein type was set as “*Homo sapiens*”, the confidence was set to >0.9, and the other parameters remained the default, to obtain the protein interaction relationship. The results were imported into Cytoscape 3.9.0 software, and the key genes of TOP10 were screened by the “MCC” algorithm with high prediction accuracy for key proteins in the PPI network in the “CytoHubba” plug-in. The GEPIA database (http://gepia.cancer-pku.cn/) was used to verify the mRNA expression levels of the selected key genes in HCC and normal liver tissues, and the total survival time of 10 key genes overexpression in HCC patients was analyzed, with 50% up and downregulated of gene expression as the analysis standard. In this study, HCC patients were divided into two groups according to the median expression of core genes. Log-rank *p* < 0.05 was considered statistically significant. The protein expression levels of key genes in normal liver tissues and HCC tissues were detected by The Human Protein Atlas (HPA) database (https://www.proteinatlas.org/). Finally, the mRNA and protein expression levels in HCC and normal liver tissues were different, and the key genes with statistically significant overall survival in HCC patients were used as the core genes for further analysis.

### GO functional enrichment analysis and KEGG pathway analysis

The gene information of potential targets for drug treatment of hepatocellular carcinoma was analyzed using the “Clusterprofiler” package of R software for GO functional enrichment and KEGG pathway analysis. *p* < 0.05 was used as the screening index (*p* < 0.01 was considered significant enrichment, and the smaller the *p*-value was, the more significant the enrichment).

### Drug similarity and ADME characteristics analysis

There are many compounds in traditional Chinese medicine, and *Scutellaria barbata* is orally taken after decoction in traditional clinical use. Therefore, combined with clinical application, the ingredients absorbed by oral administration are the effective ingredients of *Scutellaria barbata* in the treatment of hepatocellular carcinoma. Ideal oral drug molecules should conform to Lipinski’s five rules of physical and chemical properties; which predicts the drug similarity of a compound with certain biological activity. According to Lipinski’s five rules, drug-like compounds used for oral administration should exhibit the following characteristics: MW ≤ 500; mlogP ≤4.5; number of rotatable bonds ≤5; hydrogen bond acceptors <10; and hydrogen bond donor <5; The Swiss ADME network service platform (http://www.swissadme.ch/)) was used to analyze the ADME characteristics of TCM ingredients interacting with the core target genes according to the ingredient-target interaction information in the “Targets Information” in the “Related Targets” section of TCMSP database. Because the expected action site of the screened compounds is the liver, it is hoped that the active ingredients should not penetrate the blood-brain barrier (BBB permeant) to reduce the adverse reactions caused by the later development of active ingredients. Finally, compounds conforming to the Lipinski's five rules and not penetrating the BBB were selected for further molecular docking with the interacting target proteins.

#### Molecular docking verification of active ingredients with target proteins

The 3D structure of the compound was downloaded from the Pubchem database, and Chem3D2019 software was used for energy optimization to obtain the 3D structure of the compound, which was stored in mol2 format. The 3D crystal structure of the target protein with resolution below 2.5 Å was download from the PDB (https://www.rcsb.org/) database and saved it in PDB format. Water molecules and small molecular ligands were removed in PyMOL2.5.2 software. The MGLTools 1.5.7 support tool of Audock vina software is used to hydrogenate the corresponding, complete the selection of receptors, and set the docking active center (including the entire protein molecule) through the grid box function in the software. Finally, the docking was completed by vina molecular docking function to verify the screening results of network pharmacology. The smaller the molecular binding energy (affinity) is, the better the binding activity between the protein and core active ingredients.

## Cell lines and culture

Human hepatocellular carcinoma cell HepG2 and human normal Hep3B were cultured in Minimum Essential Medium (MEM) containing 10% fetal bovine serum (FBS), streptomycin (100 ng/ml), and penicillin (100 U/ml), cells and culture medium from Zhong Qiao Xin Zhou Biotechnology. The parameters of the incubator were 5% CO2 and 37°C.

### Compound

Rhamnazin (CMEC) and Luteolin (CMEC) dissolution with DMSO. When used, diluted with complete medium to a specific concentration (DMSO <0.05%) drug-containing solution.

#### Cell viability assay

Cells were inoculated onto 96-well plates at 1 × 105/ml (100ul/well) and cultured overnight. CCK-8 kit (proteinbio) was used to cell viability detection. Cells treated with drug-containing medium for 24H. Remove the original medium and wash twice with 100ul PBS per well. Add 100ul complete medium (containing 10ul CCK-8 kit) to each well), Incubation of 2H, then use of enzyme calibration (Biotek synergy HI) detection OD value at 450 nm wavelength.

### Statistical analysis

The data are presented as the means ± standard deviations (SD). GraphPad Prism software was used to draw graphs. The comparison among groups were tested using one-way analysis of variance (ANOVA). The Student’s t-test was used to analyze the differences between two groups. *p* < 0.05 was considered statistically significant.

## Results

### Screening results of active ingredients and targets of *Scutellaria barbata*

According to the criteria of oral bioavailability (OB) ≥ 30% and drug-like (DL) ≥ 0.18, 29 active ingredients of *Scutellaria barbata* were screened from the TCMSP database (Table 1). A total of 461 active ingredient-related targets were collected through the TCMSP database and Swiss Target Prediction network service platform.

**TABLE 1:** Active constituents of *Scutellaria barbata*.

Mol ID	Molecule name	OB (%)	DL
MOL001040	(2R)-5,7-dihydroxy-2-(4-hydroxyphenyl)chroman-4-one	42.36	0.21
MOL012245	5,7,4′-trihydroxy-6-methoxyflavanone	36.63	0.27
MOL012246	5,7,4′-trihydroxy-8-methoxyflavanone	74.24	0.26
MOL012248	5-hydroxy-7,8-dimethoxy-2-(4-methoxyphenyl)chromone	65.82	0.33
MOL012250	7-hydroxy-5,8-dimethoxy-2-phenyl-chromone	43.72	0.25
MOL012251	Chrysin-5-methylether	37.27	0.20
MOL012252	9,19-cyclolanost-24-en-3-ol	38.69	0.78
MOL002776	Baicalin	40.12	0.75
MOL012254	campesterol	37.58	0.71
MOL000953	CLR	37.87	0.68
MOL000358	beta-sitosterol	36.91	0.75
MOL012266	rivularin	37.94	0.37
MOL001973	Sitosteryl acetate	40.39	0.85
MOL012269	Stigmasta-5,22-dien-3-ol-acetate	46.44	0.86
MOL012270	Stigmastan-3,5,22-triene	45.03	0.71
MOL000449	Stigmasterol	43.83	0.76
MOL000173	wogonin	30.68	0.23
MOL001735	Hispidulin	30.97	0.27
MOL001755	24-Ethylcholest-4-en-3-one	36.08	0.76
MOL002714	baicalein	33.52	0.21
MOL002719	6-Hydroxynaringenin	33.23	0.24
MOL002915	Salvigenin	49.07	0.33
MOL000351	Rhamnazin	47.14	0.34
MOL000359	sitosterol	36.91	0.75
MOL005190	eriodictyol	71.79	0.24
MOL005869	daucostero_qt	36.91	0.75
MOL000006	luteolin	36.16	0.25
MOL008206	Moslosooflavone	44.09	0.25
MOL000098	quercetin	46.43	0.28

Results of target collection for hepatocellular carcinoma.

By using “hepatocellular carcinoma” and “HCC” as keywords, 53 targets were collected in the TTD database, 7917 targets in the GeneCards database, 559 targets in the Disgenet database and 236 targets in the OMIM database. After the repetition was removed, 8238 potential therapeutic targets for HCC were obtained.

### Target of Scutellaria barbata for hepatocellular carcinoma

**FIGURE 1:**
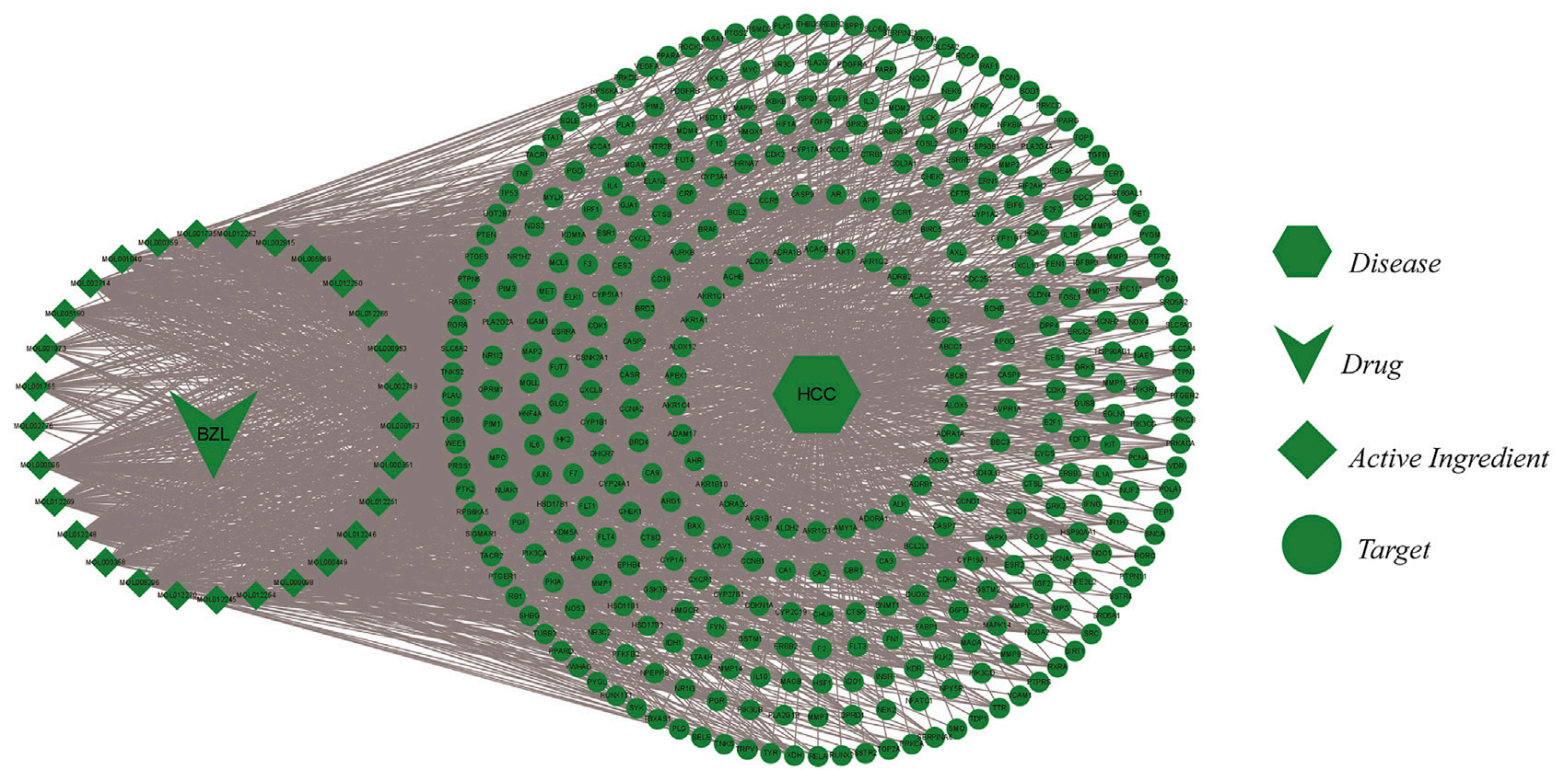
“Chinese medicine-active ingredient—Target-disease” regulatory network.

A total of 372 potential therapeutic targets for hepatocellular carcinoma were obtained by crossing the targets of active ingredients and potential therapeutic targets of hepatocellular carcinoma. Cytoscape software was used to construct the “Chinese medicine-ingredient—target-disease” regulatory network (Figure 1). The control network has 403 nodes and 2424 edges.

### PPI network construction and core gene screening and verification

**FIGURE 2:**
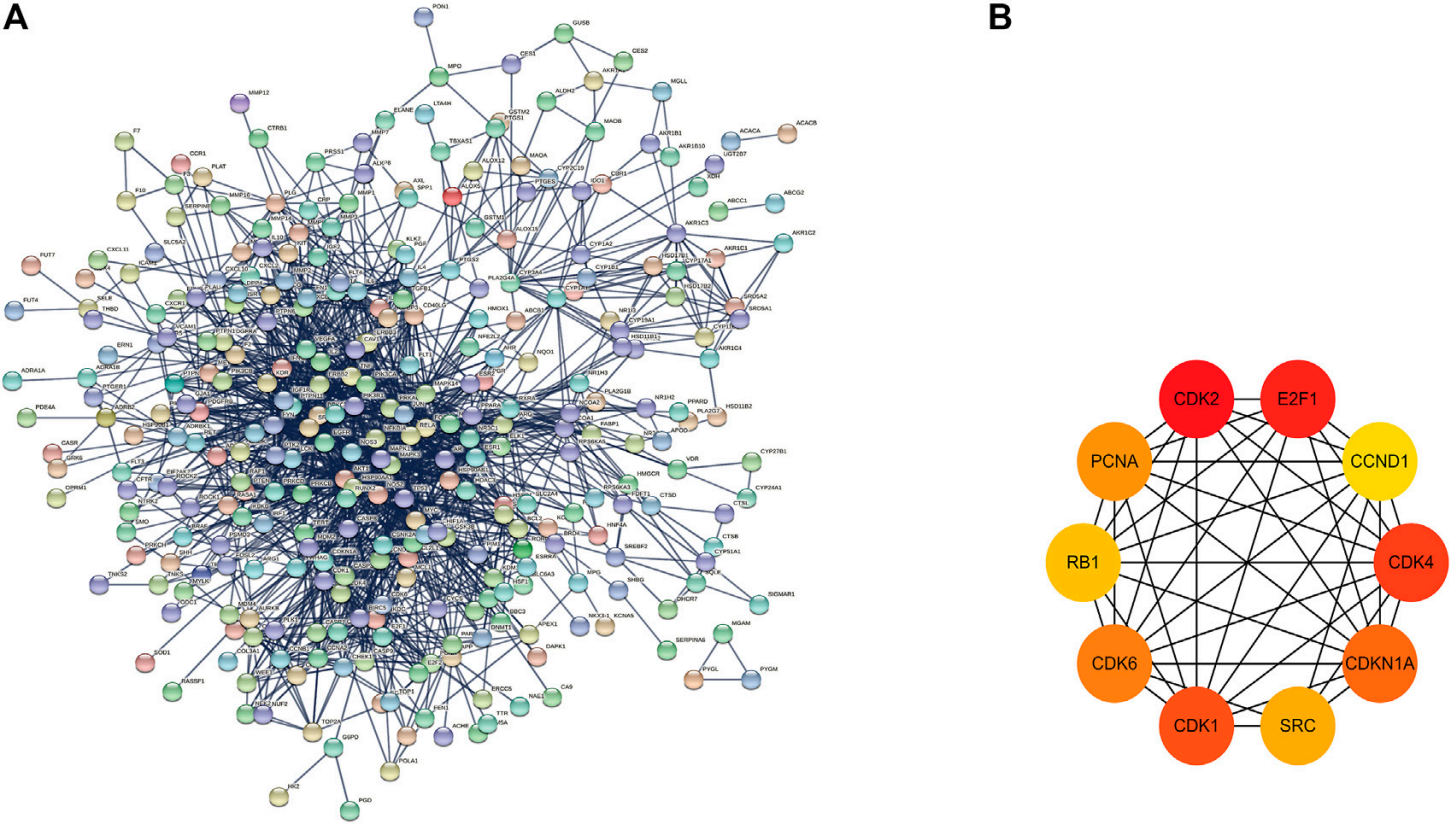
Drug-disease cross-target protein interaction network **(A)** and key gene module **(B)**.

**FIGURE 3:**
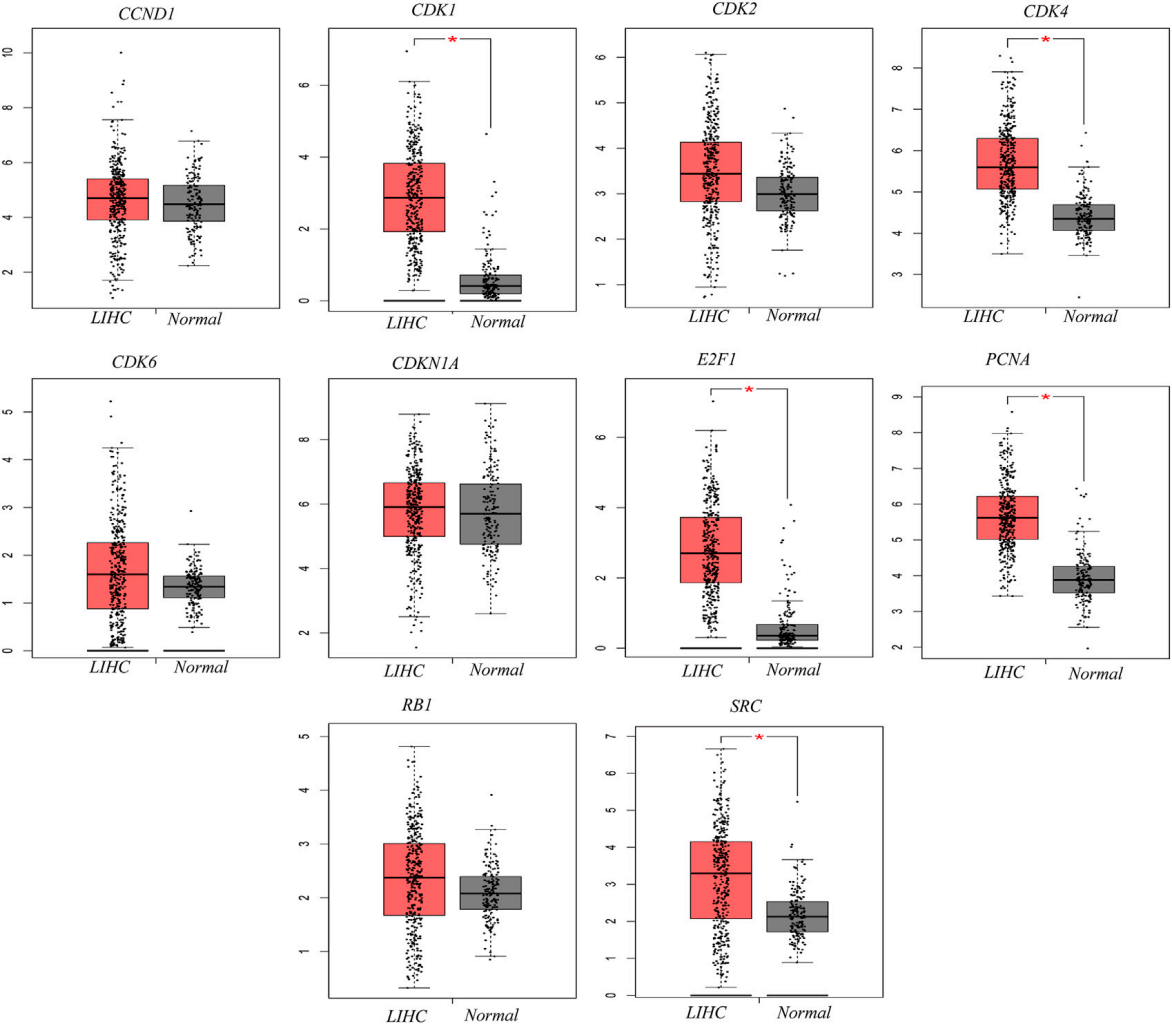
Validation of mRNA expression of key genes in LIHC and normal liver tissues by GEPIA database (The 10 box diagrams were based on 369 HCC samples (red markers) and 160 normal samples (gray markers). *p < 0.01 considered statistically significant).

**FIGURE 4:**
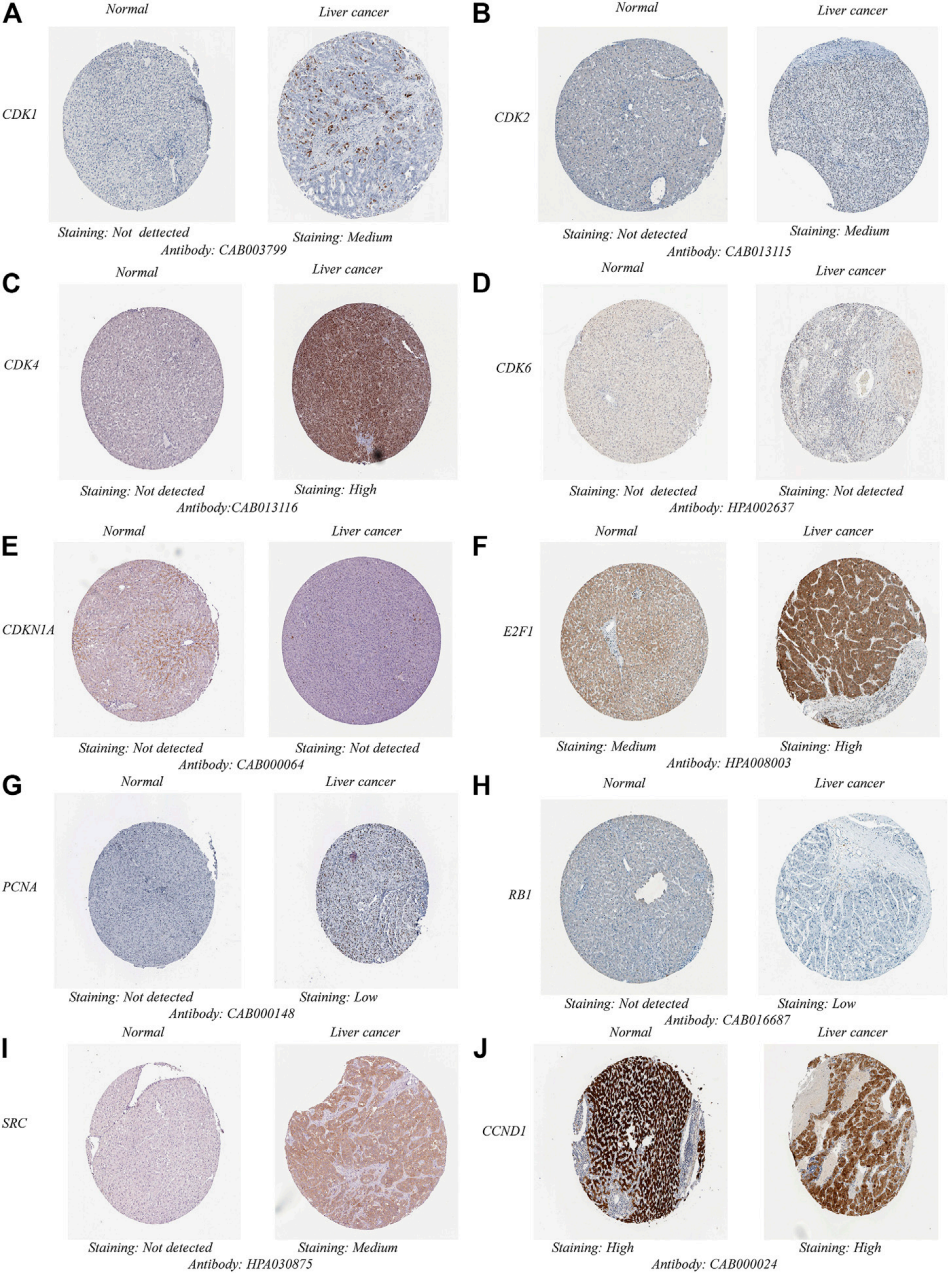
Representative immunohistochemistry images of **(A)** CDK1, **(B)** CDK2, **(C)** CDK4, **(D)** CDK6, **(E)** CDKN1A, **(F)** E2F1, **(G)** PCNA, **(H)** RB1, **(I)** SRC, and **(J)** CCND1 in HCC and non-cancerous liver tissues derived from the HPA database. HCC, hepatocellular carcinoma; HPA, Human Protein Atlas.

**FIGURE 5:**
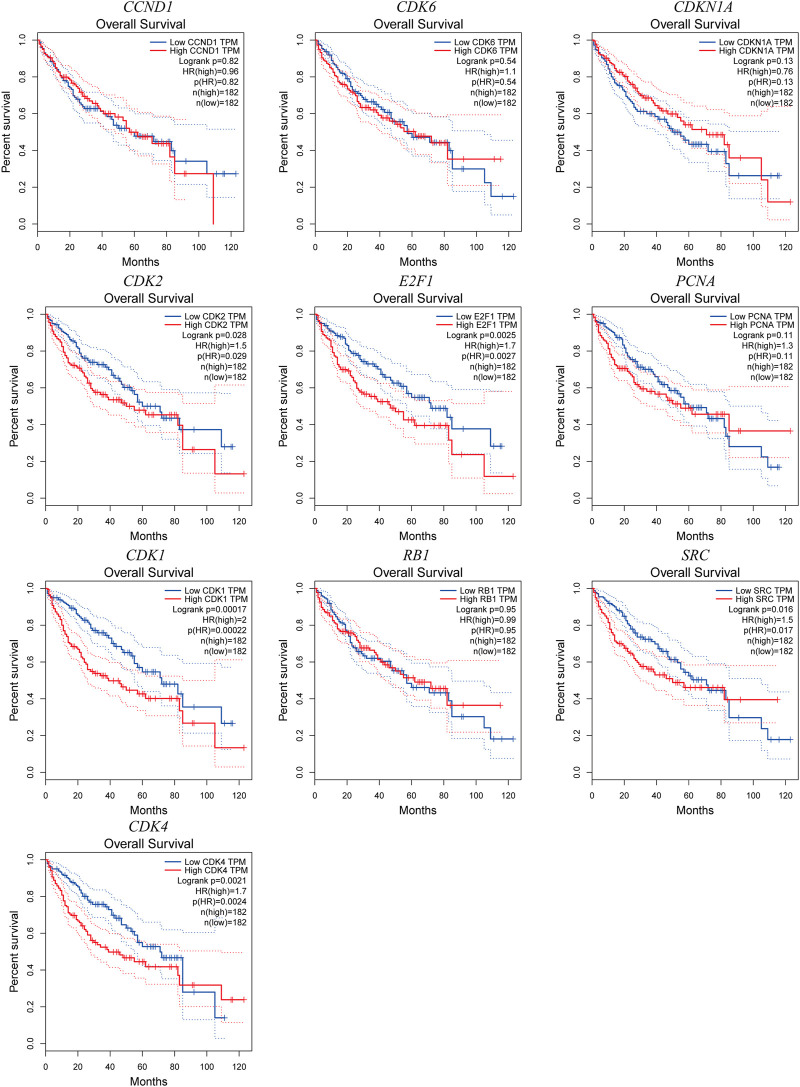
The overall survival time of 10 key genes overexpression in LIHC patients.

The potential target gene information of drug treatment diseases was imported into the STRING online platform (node with 0 hidden value), and the results were imported into Cytoscape3.9.0 software. The key genes of TOP10 were obtained by the MCC′ algorithm of′ Cytohubba′ plug-in (Figure 2). GEPIA database analysis showed that the mRNA expression levels of key genes CDK1, SRC, CDK4, PCNA and E2F1 in HCC were significantly upregulated compared with those in normal liver tissues (Figure 3). The Human Protein Atlas (HPA) results showed that the CDK4 gene was highly expressed in liver cancer tissues, the CDK1, SRC, and CDK2 genes were moderately expressed in liver cancer tissues, and the PCNA, and RB1 genes were lowly expressed in liver cancer tissues, but not expressed in normal liver tissues, the CCND1 gene was highly expressed in liver cancer tissues and normal liver tissues. The E2F1 gene was moderately expressed in normal liver tissue and highly expressed in liver cancer tissue; The CDKN1A and CDK6 were not expressed in liver cancer tissues or normal liver tissues (Figure 4). The results of GEPIA survival analysis showed that the high expression of the key genes CDK1, CDK2, CDK4, E2F1, and SRC in HCC was correlated with poor prognosis (Log-rank *p* < 0.05) (Figure 5). Combined with the verification results of key genes, it was found that the mRNA and protein expression levels of CDK1, SRC, CDK4, and E2F1 were overexpressed in HCC tissues compared with those in normal liver tissues, and the high expression in HCC patients was related to poor prognosis. These results revealed that CDK1, SRC, CDK4, and E2F1 may be the core genes of *Scutellaria barbata* to target in the treatment of HCC.

### GO and KEGG enrichment analysis results

**FIGURE 6:**
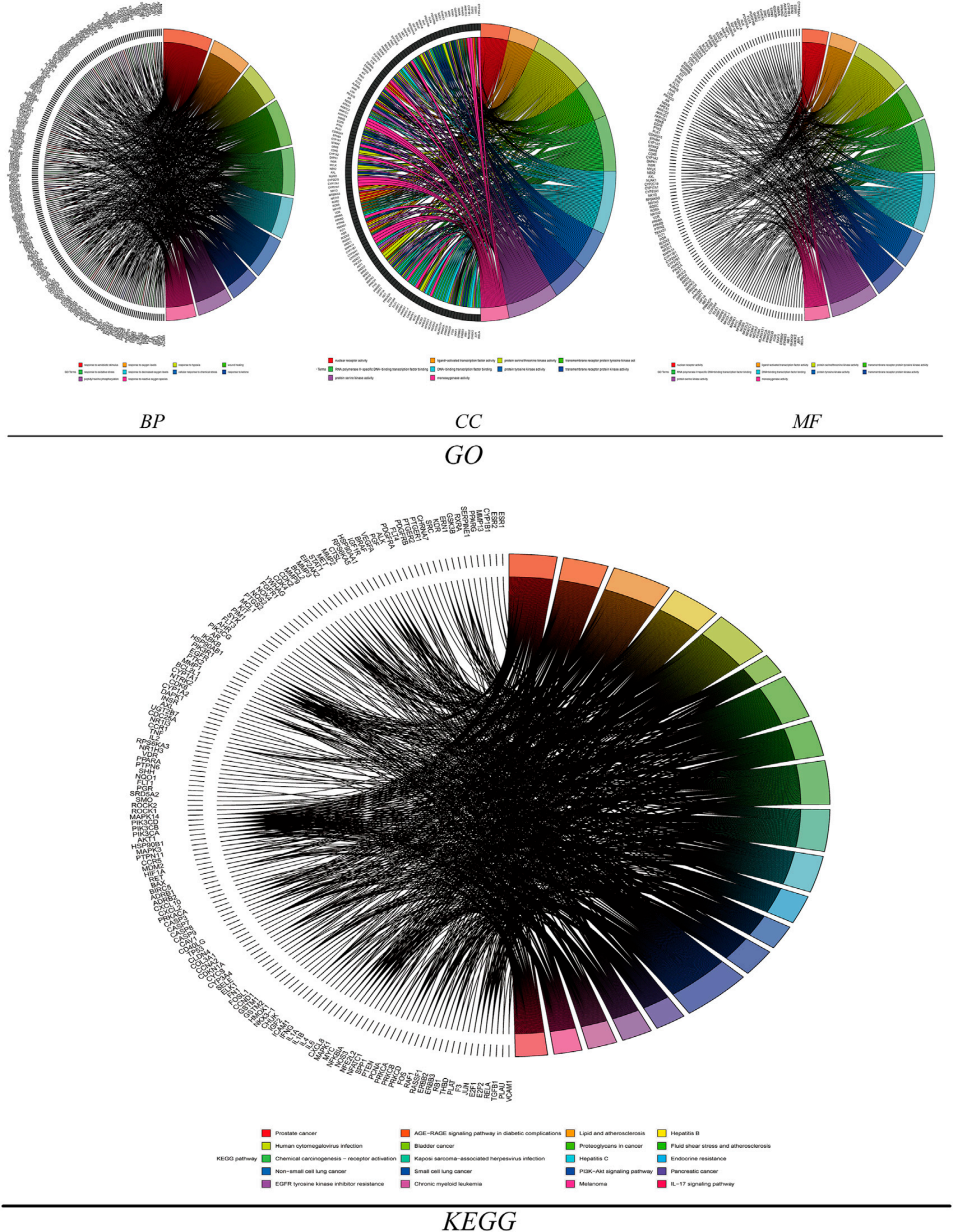
GO enrichment analysis and KEGG enrichment analysis (BP: biological process, CC: cell ingredient, MF: molecular function).

The gene information of potential targets for drug treatment of hepatocellular carcinoma was used for GO and KEGG enrichment analysis using the “ClusterProfiler” package of R software. A total of 3056 GO enrichment entries were obtained, including biological processes (BP, 2717), cell components (CC, 91), and molecular functions (MF, 248). The main enriched biological processes included response to xenobiotic stimuli, wound healing, response to oxidative stress; the main enriched cell components were plasma membrane raft, vesicle lumen, organelle outer membrane. The main enriched molecular functions included nuclear receptor activity, ligand-activated transcription factor activity, transmembrane receptor protein tyrosine kinase activity, etc. The KEGG pathway analysis was enriched to 180 enrichment results. The results of KEGG pathway enrichment analysis showed that genes were mainly enriched in the PI3K-Akt signaling pathway, IL-17 signaling pathway, TNF signaling pathway, apoptosis and other pathways in addition to various cancer pathways. The 10 pathways with the smallest *p* values of biological processes, cell components and molecular functions in GO enrichment analysis and the *p* values of KEGG enrichment analysis were the highest based on analysis using R language software (Figure 6).

### Drug similarity and ADME characteristics analysis

According to the “ingredient-target” interaction information of “Targets Information” in the “Related Targets” section of the TCMSP database, one ingredient interacting with CDK1, SRC, and E2F1, nine ingredients interacting with CDK1 and SRC, and one ingredient interacting with CDK4 and CDK1 in *Scutellaria barbata* was found. There were five ingredients that only interacted with SRC and one ingredient that only interacted with CDK1. There are 17 ingredients interacted with core gene targets in *Scutellaria barbata*. The Swiss ADME network service platform was used to screen 12 compounds that conformed to the five rules of Lipinski and did not penetrate the BBB (Table 2).

**TABLE 2:** ADME characteristics of core active ingredients.

Molecule ID	MW	Rotatable bonds	H-bond acceptors	H-bond donors	MlogP	BBB permeant	Lipinski violations
MOL012245	302.28	2	6	3	0.41	No	0
MOL012246	302.28	2	6	3	0.41	No	0
MOL002719	288.25	1	6	4	0.16	No	0
MOL002714	270.24	1	5	3	0.52	No	0
MOL001735	300.26	2	6	3	0.22	No	0
MOL005190	288.25	1	6	4	0.16	No	0
MOL000006	302.24	1	7	4	−0.56	No	0
MOL000098	302.24	1	7	5	−0.56	No	0
MOL012266	344.32	4	7	2	0.17	No	0
MOL000173	284.26	2	5	2	0.77	No	0
MOL000351	330.29	3	7	3	−0.07	No	0
MOL001040	272.25	1	5	3	0.71	No	0

Molecular docking results.

**FIGURE 7:**
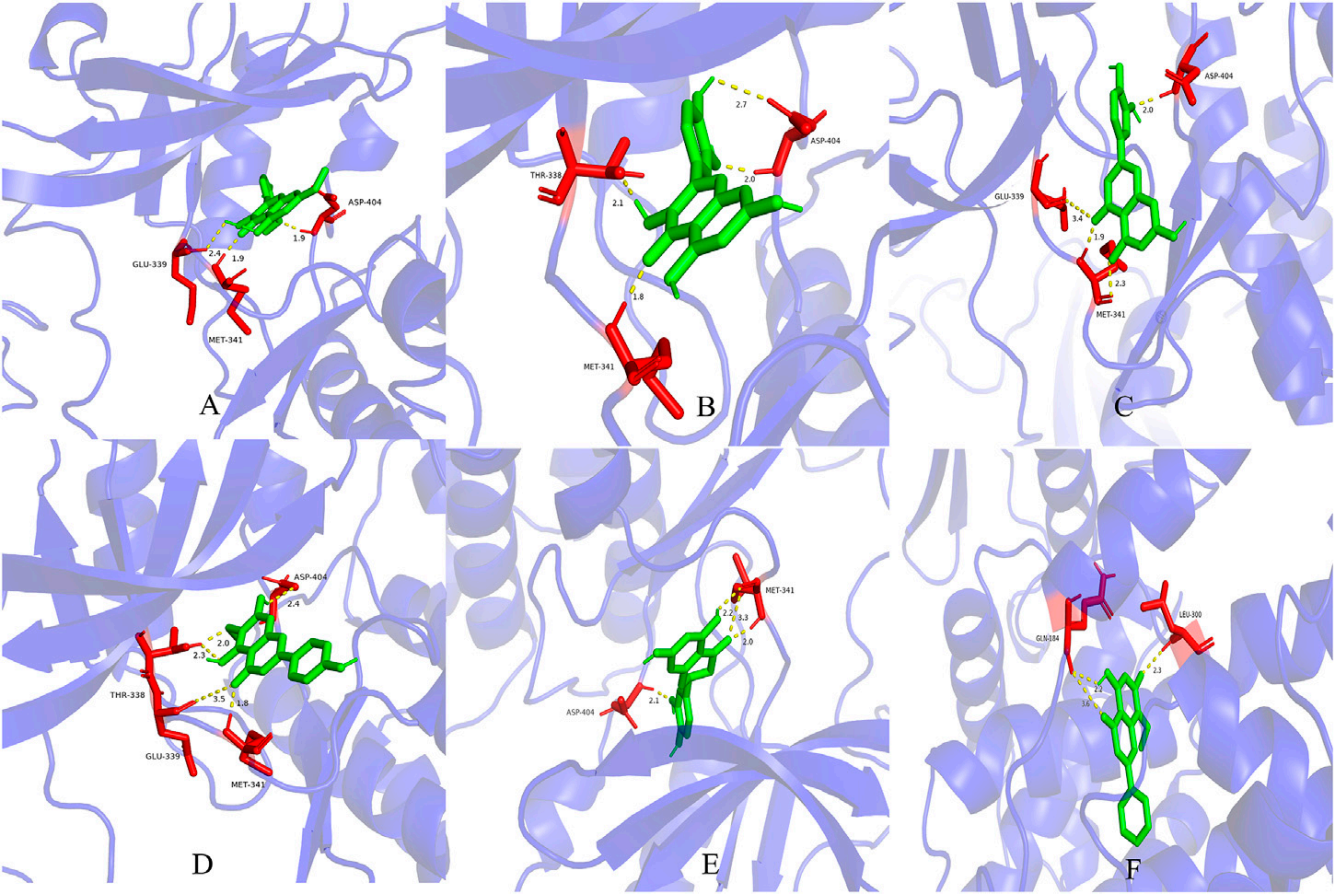
Molecular docking mode of the first six compounds with the lowest Vina score **(A)**:SRC**-** Rhamnazin; **(B)** SRC- quercetin; **(C)** SRC- luteolin; **(D)** SRC-Hispidulin; **(E)** SRC- eriodictyol; **(F)** CDK1- Wogonin).

AutoDock Vina software was used for molecular docking of the core gene target protein and the interaction of traditional Chinese medicine ingredients. It is generally believed that the lower the binding energy of the ligand and receptor, the better the binding force. When the binding energy is less than 0 kcal/mol, the two can spontaneously combine. When the binding energy is less than −5 kcal/mol, the binding force is better. When the binding energy is less than −7 kcal/mol, the binding configuration has strong activity. According to the molecular docking results, the binding energy of all the core active ingredients with the core target protein was ≤ −5 kcal/mol (Table 3). The docking modes of the first six compounds with the lowest Vina score were visualized (Figure 7).

**TABLE 3:** Molecular docking energy.

Target	PDB ID	MOL ID	Compound	Aniffnity (kcal/mol)
SRC	2SRC	MOL000351	Rhamnazin	−9.5
SRC	2SRC	MOL000098	quercetin	−9.2
SRC	2SRC	MOL000006	luteolin	−9
SRC	2SRC	MOL001735	Hispidulin	−8.9
SRC	2SRC	MOL005190	eriodictyol	−8.9
CDK1	6GU2	MOL000173	wogonin	−8.8
SRC	2SRC	MOL002714	baicalein	−8.8
SRC	2SRC	MOL012245	5,7,4′-trihydroxy-6-methoxyflavanone	−8.8
SRC	2SRC	MOL000173	wogonin	−8.6
SRC	2SRC	MOL001040	(2R)-5,7-dihydroxy-2-(4-hydroxyphenyl)chroman-4-one	−8.6
CDK4	2W96	MOL012246	5,7,4′-trihydroxy-8-methoxyflavanone	−8.6
SRC	2SRC	MOL002719	6-Hydroxynaringenin	−8.5
CDK1	6GU2	MOL000006	luteolin	−8.4
CDK1	6GU2	MOL000351	Rhamnazin	−8.4
CDK1	6GU2	MOL001735	Hispidulin	−8.3
CDK1	6GU2	MOL002714	baicalein	−8.3
CDK1	6GU2	MOL000098	quercetin	−8.2
CDK1	6GU2	MOL012246	5,7,4′-trihydroxy-8-methoxyflavanone	−8.1
SRC	2SRC	MOL012266	rivularin	−7.9
E2F1	6G0P	MOL000098	quercetin	−6.4

### Rhamnazin and Luteolin inhibited the proliferation of HepG2 cells and Hep3B cells

**FIGURE 8:**
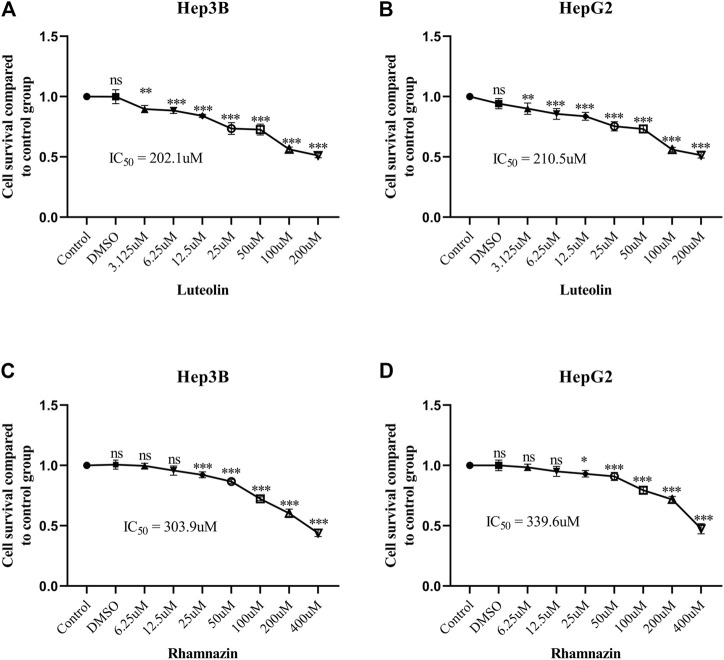
Rhamnazin and Luteolin inhibits cell viability. **(A, B)** Viability of Hep3B and HepG2 after being treated with the indicated doses of Luteolin, respectively. **(C, D)** Viability of Hep3B and HepG2 after being treated with the indicated doses of Rhamnazin, respectively. All data are presented as mean ± standard deviation (SD; *n* = 5). (*: *p* < 0.05, **: *p* < 0.01, ***: *p* < 0.001).

After adding different concentrations of drugs to treat HepG2 cells and Hep3B cells, CCK-8 kit was used to detect cell activity. The experimental results showed that specific concentrations of Rhamnazin and Luteolin could inhibit the proliferation of HepG2 cells and Hep3B cells (Figure 8).

## Discussion

29 potential active ingredients and 372 potential targets of *Scutellaria barbata* in the treatment of hepatocellular carcinoma were preliminarily screened. Through the construction of a protein interaction network and screening and verification of core targets, it was found that CDK1, SRC, CDK4 and E2F1 mRNA and protein expression levels were overexpressed in liver cancer tissues compared with normal liver tissues. Survival analysis showed that these genes were highly expressed in liver cancer patients and correlated with poor prognosis. According to previous studies, miR-203 inhibits the proliferation and migration of lung cancer cells and promotes apoptosis of lung cancer cells by targeting SRC expression (Wang et al., 2014). CDK1 is a typical cell cycle checkpoint involved in cell proliferation and transcription regulation (Asghar et al., 2015); it is essential for cell cycle progression to M phase, which is related to cyclin A and B, respectively, and regulates the transformation of S-G2 and G2-M. *In vitro* and *in vivo* studies, cell cycle progression was blocked by downregulate CDK1 in liver cancer cells in the G2-M phase (Zhao et al., 2013; Nguyen et al., 2016). Metformin induces mir-378 to down-regulate CDK1, resulting in inhibition of proliferation of hepatocellular carcinoma cells (Zhou et al., 2018). miR-1271 overexpression inhibits the proliferation of endometrial cancer cells and induces apoptosis by targeting CDK1 (Li et al., 2017). CDK4 specifically regulates the cell cycle transition from G1 phase to S phase. CDK4 inhibitors effectively block the proliferation of cancer cells by inducing G1 phase cell cycle arrest (Goel et al., 2018). By knocking out lncRNA LOC90784, CDK4 expression is inhibited, thereby inhibiting the proliferation of liver cancer cells, and inducing apoptosis and cell cycle arrest (Xu et al., 2017). E2F1 is involved in the cell cycle and DNA synthesis by regulating the expression of cell cycle-related genes in eukaryotic cells (Tazawa et al., 2007). The abnormal expression of E2F1 in hepatocellular carcinoma leads to tumor cell proliferation and invasion (Gu et al., 2018). MicroRNA-34a inhibits the invasiveness of hepatocellular carcinoma by inhibiting the expression of E2F1 (Han et al., 2019). Celastrol induces apoptosis and inhibits the proliferation of hepatocellular carcinoma cells by downregulating E2F1 (Ma et al., 2017).

The screened standard active compounds were confirmed in previous studies, and the combination of wogonin and sorafenib effectively killed human liver cancer cells by enhancing cell apoptosis and inhibiting autophagy (Rong et al., 2017). wogonin induces autophagy, apoptosis and G2/M cell cycle arrest in human colorectal cancer cells by inhibiting the PI3K-AKT signaling pathway, and inhibits the proliferation of human colorectal cancer cells (Tan et al., 2019). Rhamnazin has antiangiogenesis activity and antitumor effect (Yu et al., 2015). Hispidulin induces cell death in a dose- and time-dependent manner in HepG2 cells, while no toxic reaction at a certain concentration is observed in normal hepatocytes. Hispidulin induces apoptosis of HepG2 cancer cells by inducing mitochondrial dysfunction and inhibiting P13k-Akt signaling pathway (Gao et al., 2014). Luteolin has been proved to have anticancer activity, including inducing apoptosis, cell cycle arrest and antiangiogenesis (Hussain et al., 2021). Luteolin and sorafenib were combined to kill human hepatocellular carcinoma cells by enhancing apoptosis and JNK activation (Feng et al., 2018). Baicalein has a variety of antihepatoma effects, including arresting cell cycle, inhibiting cell proliferation, inhibiting migration, inducing apoptosis, and inducing autophagy by regulating related molecules and signaling pathways (Bie et al., 2017). Quercetin has obvious antiproliferative and proapoptotic effects in HCC (Fernández-Palanca et al., 2019). Eriodictyol inhibits proliferation, and metastasis and induces apoptosis of glioma cells by blocking PI3K-Akt signaling pathway (Li et al., 2020).

The above studies demonstrate that the core genes are related to the occurrence and progression of cancer, active ingredients have anticancer effects, and some active ingredients have been shown to inhibit the proliferation and migration of liver cancer cells and induce apoptosis, which demonstrates the scientific nature of this study. Therefore, combined with the analysis results, it is considered that *Scutellaria barbata* may play a role in the treatment of HCC through the following aspects.

### Interaction of ‘active ingredient-core target'

Previous studies have shown that inhibiting the expression of core target genes can inhibit the proliferation of cancer cells and induce apoptosis of cancer cells. According to the results of survival analysis, the high expression of these genes in hepatocellular carcinoma is related to poor prognosis. The molecular docking results of the above core targets and their corresponding active ingredients show good active configurations. Combined with previous studies on the role of core genes in the occurrence and progression of cancer and the pharmacological effects of active ingredients, it is speculated that the mechanism of *Scutellaria barbata* in the treatment of hepatocellular carcinoma may be through Rhamnazin and Hispidulin etc. Active ingredients such as baicalein inhibit the expression of core genes, regulate the cell cycle, inhibit the proliferation and migration of cancer cells, and induce apoptosis.

### Blocking the PI3K-AKT signaling pathway

The PI3K-Akt signaling pathway plays an important role in the results of KEGG enrichment analysis. The PI3K-Akt signaling pathway is closely related to cell proliferation, cell cycle progression and apoptosis (Noorolyai et al., 2019). Inhibition of HCC progression occurs through inhibition of the PI3K-Akt signaling pathway (Liu et al., 2021). Combined with the results of network pharmacology analysis and previous studies, it is speculated that *Scutellaria barbata* may block the PI3K-Akt signaling pathway by active components such as wogonin, hispidulin and eriodictyol etc to inhibit the proliferation and migration of cancer cells, induce apoptosis of cancer cells, and play a role in the treatment of hepatocellular carcinoma.

Combined with the results of network pharmacology analysis and previous studies, it is speculated that *Scutellaria barbata* may block the PI3K-Akt signaling pathway by active ingredients such as wogonin, hispidulin and hriodictyol etc to inhibit the proliferation and migration of cancer cells, induce apoptosis of cancer cells, and play a role in the treatment of hepatocellular carcinoma.

In this study, through the network pharmacology method to determine the core target, further screening in liver cancer tissue and normal liver tissue showed that the mRNA and protein expression levels were different, and abnormal gene expression and poor prognosis-related genes were identified in hepatocellular carcinoma patients for follow-up analysis. In addition to the clinical characteristics of *Scutellaria barbata,* used mainly for oral administration for the treatment of hepatocellular carcinoma in this study, the AMDE characteristics of the active ingredients of *Scutellaria barbata* were analyzed, and were in accordance with the principle of oral administration and not through the BBB and were screened to reduce the adverse reactions after later development. Finally, the further screened genes and the required active ingredients were combined with network pharmacology analysis and previous research results to explore the mechanism of *Scutellaria barbata* in the treatment of hepatocellular carcinoma and improve the accuracy and scientificity of the analysis results. The results of this study are mainly based on the network pharmacology method. Currently, some active ingredients screened have no pharmacological effects and are limited to the database, so there may be some differences in the actual action process of *Scutellaria barbata*.

## Conclusion

In this study, combined with the practical application of *Scutellaria barbata* in clinical practice, the network pharmacology method and molecular docking technology were used to analyze the mechanism of active ingredients in accordance with oral drug-like rules in the treatment of hepatocellular carcinoma. A preliminary theoretical study was carried out to explore the development of active ingredients of *Scutellaria barbata* for HCC treatment. We expeect to provide more options for the clinical treatment of hepatocellular carcinoma.

## Data Availability

The original contributions presented in the study are included in the article/supplementary material, further inquiries can be directed to the corresponding author.

## Abbreviations

HCC, Hepatocellular carcinoma; TCM, Traditional Chinese medicine; OB, Oral bioavailability; DL, Drug-like index; HPA, Human protein map; GO, Gene ontology; KEGG, Kyoto encyclopedia of genes and GeNomes; BP, Biological processes; CC, Cellular components; MF, Molecular function; PPI, Protein-protein interaction; AMDE, Absorption, distribution, metabolism, excretion; BBB, Blood-brain barrier.
